# Prognostic and Predictive Relevance of Tumor-Infiltrating Lymphocytes in Squamous Cell Head–Neck Cancer Patients Treated with Radical Radiotherapy/Chemo-Radiotherapy

**DOI:** 10.3390/curroncol29060342

**Published:** 2022-06-15

**Authors:** Ioannis M. Koukourakis, Anastasia G. Gkegka, Erasmia Xanthopoulou, Christos Nanos, Alexandra Giatromanolaki, Michael I. Koukourakis

**Affiliations:** 1 1st Department of Radiology, Radiation Oncology Unit, Aretaieion University Hospital, 15772 Athens, Greece; koukourioannis@gmail.com; 2 Department of Pathology, Medical School, Democritus University of Thrace, 68100 Alexandroupolis, Greece; linda03red@hotmail.com (A.G.G.); agiatrom@med.duth.gr (A.G.); 3 Department of Radiotherapy/Oncology, Medical School, Democritus University of Thrace, 68100 Alexandroupolis, Greece; mia_x1995@hotmail.com (E.X.); c_nanos@hotmail.com (C.N.)

**Keywords:** squamous cell head–neck cancer, radiotherapy, tumor-infiltrating lymphocytes, hypoxia, prognosis

## Abstract

Microenvironmental conditions control the entrance and thriving of cytotoxic lymphocytes in tumors, allowing or preventing immune-mediated cancer cell death. We investigated the role of tumor-infiltrating lymphocyte (TIL) density in the outcome of radiotherapy in a series of squamous cell head–neck tumors (HNSCC). Moreover, we assessed the link between markers of hypoxia and TIL density. One-hundred twenty-one patients with HNSCC treated prospectively with radical radiotherapy/chemo-radiotherapy were analyzed. The assessment of TIL density was performed on hematoxylin and eosin biopsy sections before radiotherapy. TIL density ranged from 0.8 to 150 lymphocytes per ×40 optical field (median 27.5). Using the median value, patients were grouped into two categories of low and high TIL density. Early T-stage tumors had a significantly higher TIL density (*p* < 0.003), but we found no association with N-stage. Overexpression of HIF1α, HIF2α, and CA9 was significantly linked with poor infiltration by TILs (*p* < 0.03). A significant association of high TIL density with better disease-specific overall survival and improved locoregional relapse-free survival was noted (*p* = 0.008 and 0.02, respectively), which was also confirmed in multivariate analysis. It is concluded that HNSCC phenotypes that allow for the intratumoral accumulation of lymphocytes have a better outcome following radical radiotherapy/chemo-radiotherapy. Intratumoral-activated HIF- and CA9-related pathways characterize immunologically cold tumors and may be used as targets for therapeutic interventions.

## 1. Introduction

Squamous cell head–neck cancer (HNSCC) is a common human tumor that is directly linked to the consumption of tobacco and alcohol. Depending upon demographic region, infection by the human papillomavirus (HPV) is also involved in the etiology of a large fraction of oropharyngeal carcinomas [1]. Radiotherapy (RT) has a major role in the management of HNSCC patients. Locally advanced tumors are most often treated with radical chemo-radiotherapy (chemo-RT), while RT is also used in early stages as monotherapy or postoperative adjuvant therapy [2,3]. With the exception of hypopharynx, the overall efficacy of surgery and chemo-RT in non-metastatic HNSCC is high in the early stages of the disease, while the cure rates of locally advanced disease range 20–50% [4]. Moreover, despite the progress in RT technology and chemotherapy, 2-year survival has not significantly improved in the last 30 years [5].

The recent developments in immunotherapy for advanced and metastatic HNSCC [6] provide a novel therapeutic window to test the efficacy of immuno-radiotherapy (immuno-RT) in an attempt to stimulate immunogenic cancer cell death in parallel with the radiogenic tumor damage. In fact, radiation-induced damage to cancer cells enhances their recognition by cytotoxic T-cells and macrophages through activation of the interferon type I and other pathways, a phenomenon known as ‘radio-vaccination’ [7]. This may also increase immunotoxic activity against distant metastasis in the context of the so-called ‘abscopal effects’ of RT, an effect that seems to demand parallel immunotherapeutic interventions in order to be substantiated in the clinical practice [8].

Activated lymphocytes could eventually infiltrate the tumor stroma and attack cancer cells. However, intratumoral lymphopenia is a common finding in a subgroup of tumors, as the ability of lymphocytes to transmigrate through the vessels into the tumor environment and their proliferative and cytotoxic activity depends on the microenvironmental conditions developed in tumors. For example, intratumoral hypoxia and acidity strongly prevent proliferation and the cytolytic activity of lymphocytes and monocytes. Moreover, conditions related to amino acid metabolism (such as arginine and tryptophan) or ATP transformation to adenosine adversely affect the survival and proliferation of cytotoxic immune cells in tumors [9].

A microenvironment that prevents the entrance and thriving of cytotoxic lymphocytes protects tumors against immune-mediated death, which may be important in the prognosis and therapeutic outcome after chemotherapy or RT. TIL density in the tumor stroma can be easily assessed in hematoxylin and eosin tissue sections used to diagnose the disease. The current study investigates TIL density in the bioptic material of a series of HNSCCs treated with radical RT or chemo-RT. Its role in response to therapy and prognosis of patients was examined. Moreover, we assessed the link between markers of hypoxia and TIL density.

## 2. Materials and Methods

One-hundred twenty-one patients with locally advanced HNSCC treated prospectively with RT/chemo-RT were analyzed. Details on the RT and chemotherapy schedules are reported in [10]. Briefly, patients were treated with accelerated hypofractionated RT delivering 20–22 fractions of 2.7 Gy to the tumor to an EQD2 (equivalent to 2 Gy fractionation dose) of 66–80 Gy. Patients were treated either by 3D-RT or VMAT RT. The 3D-RT was delivered in a three-phase approach delivering large fields to encompass the primary tumor and neck/supraclavicular area for 14–15 daily fractions, followed by a second phase of shrink fields to the tumor and high-risk areas with 1 cm margins, and finally, a third phase that provided a booster dose to the remnant tumor. The VMAT technique was used in one phase planning, where the tumor, high-risk and low-risk neck areas were treated with different daily fractionations: 2.5–2.7 Gy per fraction for the tumor area, 2.5 Gy per fraction for high-risk neck areas and 2.1–2.2 Gy for low-risk neck areas (for a total of 22 fractions). A cone-beam CT was daily performed before each radiotherapy session.

Seventeen patients received RT only, while the rest received concurrent chemo-RT with weekly cisplatin with or without cetuximab, or RT combined with cetuximab alone. Cisplatin was administered weekly at a dose of 35 mg/m^2^ when combined with cetuximab or at 35–40 mg/m^2^ when provided as a monotherapy, depending upon tolerance. Cetuximab was administered weekly at a dose of 200–250 mg/m^2^ depending upon tolerance. Twenty-five patients also received four cycles of induction chemotherapy with docetaxel/cisplatin/5-FU. Response to treatment was radiologically evaluated (CT/MRI-scans) two months after the end of therapy and 4 to 6 months thereafter. The assessment was based on the RECIST 1.1 criteria. Complete response (CR) refers to the absence of detectable disease, partial response (PR) to tumors with >30% decrease in the sum of longest diameters. A remnant scar measuring <5% of the initial dimensions was considered a CR. Progressive disease refers to >20% increase in the longest dimension. The rest of the cases were considered stable disease. The median follow-up of patients was 24 months (range 2–120 months). The patient and disease characteristics are listed in Table 1. The study has been approved by the local Ethics and Research Committee of the University Hospital of Alexandroupolis (ΔΣ7/26-2-04, ΔΣ36/34/28-9-06, and ΕΣ11/26-11-18).

### 2.1. Assessment of TILs

TIL evaluation was performed on hematoxylin- and eosin-stained slides. The number of TILs was assessed in all high-power ×40 fields throughout the whole tissue slide, whether these resided in stroma areas or cancer cell areas. Necrotic areas were excluded. The total amount of lymphocytes was divided by the overall number of optical fields (o.f.) on the corresponding slide to provide the final score of each case (mean value of all fields). The median value of the obtained scores was used to group tumors in low- and high-TIL density categories.

### 2.2. Assessment of Hypoxia-Related Markers

Data on the expression of hypoxia-inducible factors, HIF1α and HIF2α, and of the HIF-regulated carbonic anhydrase CA9 were available from previous studies in a subset of 48 (out of 121) patients herein analyzed. The methodology of immunohistochemistry, assessment and grouping of cases has been previously reported [11,12]. Briefly, tumors with cytoplasmic expression of HIFs in more than 50% of total cancer cells were grouped as high HIF expression. High CA9 expression was defined by tumors that had membrane CA9 expression in more than 10% of cancer cells after examining the whole tumor section.

### 2.3. Statistical Analysis

The GraphPad Prism 7.0 package was used for statistical analysis and the creation of graphs. The non-parametric Mann–Whitney test (for two variables) or the Kruskal–Wallis non-parametric test with subsequent Dunn test for intergroup comparison (for multiple variables) was used to compare categorical continuous tumor variables. Disease-specific overall survival (OS), locoregional relapse-free survival (LRFS), and distant metastasis-free survival (DMFS) curves were plotted using the Kaplan–Meier method. The endpoints for the OS, LRFS, and DMFS analysis were death from cancer-related reasons; radiological and clinical; or in doubtful cases, histopathological, documentation of local or regional relapse, and the radiological documentation of distant metastasis (confirmed with PET-SCAN in cases with doubtful CT or MRI imaging), respectively. LRFS and DMFS do not show the time points of death from these events, but the time-point of documentation. The starting point for survival analysis was the day after the completion of radiotherapy. The Cox-regression backward conditional method was applied for multivariate analysis of data considered as dichotomous variables. We used a trivariate model that included the major histopathological features, thus T-stage (1–2 vs. 3–4), N-stage (0–1 vs. 2–3), and the TIL density (low vs. high). Any *p*-value below <0.05 was considered statistically significant.

## 3. Results

### 3.1. TILs and Histopathological Variables

**Figure 1:**
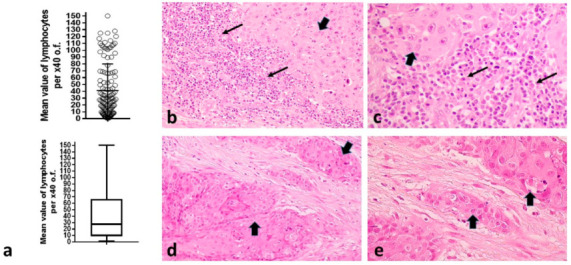
TILs in HNSCC: (**a**) Distribution of TILs per ×40 o.f. among 121 tumors (showing also the mean value and standard deviation), complemented by a box and whiskers figure showing the median value, range and the 25th and 75th percentile positions. Bars show the median value and standard deviation; (**b**,**c**) Typical hematoxylin and eosin sections of HNSCC in ×20 and ×40 magnification, respectively, showing intense infiltration of the tumor stroma by TILs; (**d**,**e**) Typical hematoxylin and eosin sections of HNSCC in ×20 and ×40 magnification, respectively, with lack of infiltration of the tumor stroma by TILs. Thin arrows show lymphocytes, while thick arrows show cancer cell nests. (HNSCC = squamous cell head–neck tumors, o.f. = optical fields, TILs = tumor-infiltrating lymphocytes).

TIL density ranged from 0.8 to 150 lymphocytes per ×40 o.f. (mean 41.2, median 27.5). A schematic representation of the distribution is shown in Figure 1a. Typical tissue sections stained for hematoxylin-eosin showing high and low infiltration of the tumor stroma by lymphocytes are presented in Figure 1b–e.

Using the median value of 27.5 (<median vs. ≥median), patients were grouped into two TIL density categories: low vs. high TIL density.

**Figure 2:**
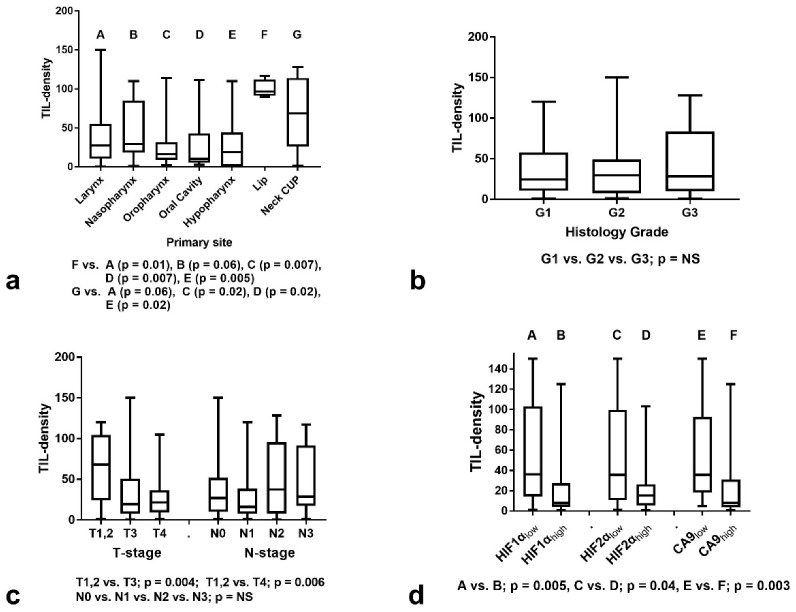
TIL density (mean value of lymphocytes per ×40 o.f.) stratified for histopathological and immunohistochemical variables: (**a**) Stratification according to primary tumor location; (**b**) Stratification according to histology grade; (**c**) Stratification according to T- and N-stage; (**d**) Stratification according to HIF1α, HIF2α, and CA9 expression groups. Box and whiskers graphs display the median value, range, and 25th and 75th percentile values. (TIL = Tumor infiltrating lymphocyte, CUP = cancer of unknown primary, G = histology grade, HIF1α = hypoxia inducible factor 1α, HIF2α = hypoxia inducible factor 2α, CA9 = carbonic anhydrase 9, A, B, C, D, E, F, G correspond to the indications shown on *x*-axis).

A graphical representation of the analysis of TIL density according to primary tumor location is shown in Figure 2a. Lip carcinoma and neck cancer of unknown primary (CUP) had significantly higher TIL density than certain other locations (Kruskal–Wallis *p* = 0.02). It must be stressed, however, that the number of lip carcinomas is too low. According to histology grade, the analysis showed no significant association with TIL density (Kruskal–Wallis *p* = 0.68) (Figure 2b).

Analysis according to T-stage showed that T1,2 cases had a significantly higher TIL density compared to T3 and T4 tumors (median ± SD: 68 ± 42 vs. 19 ± 37 vs. 21 ± 23, respectively, *p* < 0.003) (Kruskal–Wallis *p* = 0.008)–Figure 2c. Although more advanced N2,3-stages had a numerically higher TIL density (median 27 ± 36 and 16 ± 34 for N0 and N1-stage vs. 37 ± 44 and 28 ± 40 for N2 and N3-stage, respectively), the difference was not significant (*p* > 0.10) (Kruskal–Wallis *p* = 0.39)–Figure 2c.

### 3.2. TILs and Hypoxia Markers

We further examined the association of TIL density according to the expression of hypoxia-related markers. Overexpression of HIF1α, HIF2α, and CA9 was significantly linked with poor infiltration by TILs (Figure 2d). The mean ± SD TIL density was 36 ± 45, 35 ± 46, 35 ± 43 in tumors with low HIF1α, HIF2α, and CA9 expression vs. 8 ± 31, 15 ± 25, 8 ± 36 in tumors with high expression, respectively. The *p*-values were 0.005, 0.04 and 0.003, respectively.

### 3.3. TILs and Response to Therapy

**Figure 3:**
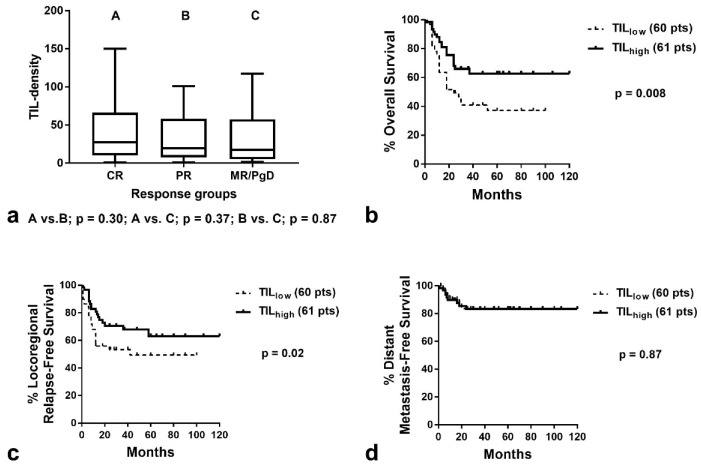
TIL density according to response to RT and Kaplan–Meier survival curves in all patients analyzed: (**a**) TIL density (box and whiskers graphs show the median value, 25th to 75th percentile and range) in patients with CR, (PR and MR/PgD; (**b**) Disease-specific overall survival according to TIL density; (**c**) Locoregional relapse-free survival according to TIL density; (**d**) Distant metastasis-free survival according to TIL density (TIL = tumor-infiltrating lymphocyte, CR = complete response, PR = partial response, MR/PgD = minimal response/progressive disease, A, B, C correspond to the indications shown on *x*-axis)).

**Figure 4:**
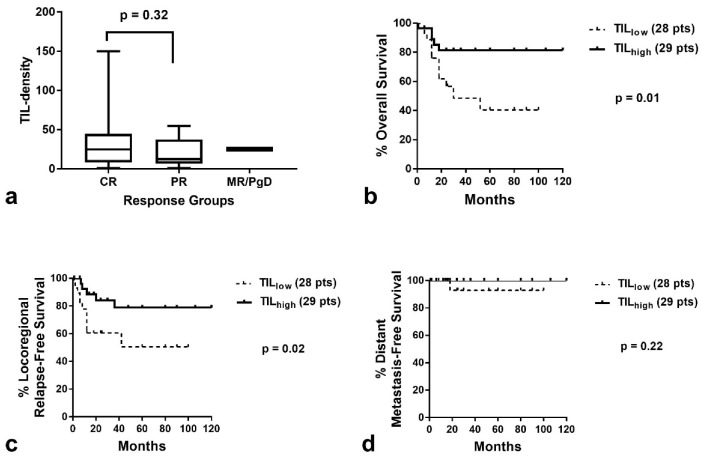
TIL density according to response to RT and Kaplan–Meier survival curves in patients with laryngeal cancer: (**a**) TIL density (median value, 25th to 75th percentile and range) in patients with CR, PR and MR/PgD; (**b**) Disease-specific overall survival according to TIL density; (**c**) Locoregional relapse-free survival according to TIL density; (**d**) Distant metastasis-free survival according to TIL density (TIL = tumor-infiltrating lymphocyte, CR = complete response, PR = partial response, MR/PgD = minimal response/progressive disease).

The median TIL density was 28.9 (range 0.9–150), 19.0 (range 0.8–101), and 17.2 (range 1.2–117) in tumors that achieved complete response, partial response, and minimal response or had progressive disease, respectively (Figure 3a). There was no statistical difference between the groups (Kruskal–Wallis *p* = 0.44). Similar results were obtained when the analysis was performed for laryngeal cancer (the largest subgroup of patients herein comprised)–Figure 4a.

### 3.4. TILs and Patient Prognosis

Kaplan–Meier survival analysis showed a significant association of high TIL density with better OS (*p* = 0.008, Hazard ratio HR = 0.46, 95% CI = 0.28–0.85–Figure 3b) and improved LRFS (*p* = 0.02, HR = 0.54, 95% CI = 0.29–0.99–Figure 3c). No association was noted with DMFS (Figure 3d). Analysis of patients with laryngeal carcinoma demonstrated similarly a significant association of high TIL density with better OS and LRFS (*p* = 0.001, HR = 0.30, 95% CI = 0.11–0.82. and *p* = 0.02, HR = 0.32, 95% CI = 0.12–0.90, respectively–Figure 4b,c). Due to the low number of patients who developed distant disease, no significant difference was noted between TIL density groups (Figure 4d).

In a trivariate Cox-regression analysis including T-stage (1/2 vs. 3/4), N-stage (0/1 vs. 2/3) and TIL density (low vs. high), nodal involvement and low TIL density were independent prognosticators of death events (*p* = 0.01, HR = 1.95, 95% CI 1.1–3.3, and *p* = 0.01, HR 2.0, 95% CI 1.1–3.6, respectively). Regarding locoregional recurrence, low TIL density was the only independent prognosticator (*p* = 0.03, HR = 1.9, 95% CI = 1.0–3.5). N-stage approached significance (*p* = 0.09, HR = 1.6, 95% CI = 0.9–3.0).

In the small group of 48 cases, where the expression of hypoxia-related molecules was available, HIF1α, HIF2α and CA9 were significantly related with poor overall survival after chemo-radiotherapy (*p* = 0.002, *p* = 0.01, and *p* = 0.04, respectively). To examine whether low TIL density defined poor prognosis separately in the low and high expression groups of HIF1α, HIF2α, and CA9, we performed a double stratification Kaplan–Meier analysis of OS, according to TIL density (low vs. high) and each one of the three hypoxia-related parameters (low vs. high). Analysis did not reveal any statistically significant associations because of the low number of cases analyzed (Supplemental Figure S1).

## 4. Discussion

The density of infiltrating lymphocytes in the tumor stroma is mainly defined by the ability of lymphocytes to enter and, subsequently, survive and proliferate in the tumor environment. Lymphocyte transmigration from vessels into the tissue stroma depends on the inflammation status of vessels, which is rather compromised in tumor vasculature; tumor vessels are often anergic to inflammatory stimuli [13]. The acidic tumor microenvironmental conditions resulting from the anaerobic usage of glycolysis are also an important factor defining poor survival, proliferation, and activity of cytotoxic T-cells [14,15]. In addition, accumulation of immunosuppressive adenosine, produced by extracellular transformation of ATP by ectonucleotidases, creates unfriendly conditions, impeding the lymphocyte from thriving in the tumor microenvironment [16]. Moreover, high levels of kynurenine (a metabolite of tryptophan) or even arginine depletion further block lymphocyte proliferation [17,18]. Thus, many vasculature- and metabolism-related conditions converge to create an adverse microenvironment for antitumor lymphocytes in a subset of tumors, namely, ‘cold tumors’ as opposed to another carcinoma group that bears intense infiltration by lymphocytes—the immunologically ‘hot tumors’.

Although the quality of TILs (regulatory vs. cytotoxic) defines the effectiveness of antitumor lymphocyte response, the density of TILs is also important in determining immune surveillance and is strongly linked with the prognosis of patients, including those with HNSCC [19]. This parameter is easily assessable in routinely used hematoxylin and eosin (H&E) tumor sections, while qualitative TIL parameters demand immunohistochemical assessment and quantification of various lymphocytic markers.

The methodology applied for quantifying TILs in H&E cancer tissue sections varies among researchers. The density of lymphocytes can be assessed in different tumor areas, such as invading front, inner stroma areas or even cancer cell nests (intra-epithelial lymphocytes) [20,21,22]. The assessment of TILs in these distinct cancer tissue areas often shows different prognostic correlations. Working groups have attempted to standardize the methodology and important guidelines have been provided [23]. The selection of a proper tissue section (or sections), followed by the selection of ‘appropriate-for-scoring’ tumor areas at low magnification, followed by determination of the type of inflammatory infiltrates (mononuclear vs. granulocytic) at higher magnification is an accepted first step. Necrotic areas and areas with superficial ulceration, often infiltrated by granulocytes, are excluded from the analysis and so is pre-existing lymphoid tissue [24,25]. However, tertiary lymphoid structures have a distinct prognostic value and should be separately assessed. When researchers focus on stroma areas, a proposed method aims to quantify subjectively or with computer image analysis the percentage of the stroma area that is covered by mononuclear cells. This method is directly related to the extent of the stroma within the tumor so that tumors with limited stroma may show high TIL scores even when a relatively low number of lymphocytes are present. Direct quantification of the number of lymphocytes per optical field in such cases, for example, would provide low TIL scores. Whether TIL assessment should be performed in selected high density areas vs. all-optical fields, in invading tumor front vs. inner tumor areas, or both, is quite unclear, but whole-slide computerized assessment followed by multiparametric analysis could resolve this problem [26]. Immunohistochemistry with pan-T and B-cell markers (such as CD3 and CD20, respectively) may better discriminate TILs among other mononuclear infiltrates. Moreover, the density of specific T-cell and monocyte subtypes seems to affect the prognostic value of TILs. Immunohistochemical sub-classification of TIL density may be important either as a prognosticator or a tool for individualized immunological phenotyping that would guide immunotherapy.

In the present study, we investigated the role of TIL density in the outcome of HNSCC patients treated with RT/chemo-RT. Since the available material was bioptic, identifying invading front vs. inner areas was not feasible. An additional problem compared to surgical material is that, quite often, insufficient presence of stroma areas hampers adequate TIL evaluation. Identifying hot spot areas may also become a source of bias in small tissue samples. Therefore, we applied a simplified method where the density of TILs was assessed in all-optical fields of the bioptic material, including stroma and cancer cell nest areas (excluding necrotic areas). The mean value was used to characterize each tumor. A large variation of TIL-densities was recorded, ranging from 0.8 to 150 lymphocytes per ×40 optical field. Advanced T-stages were statistically linked with lower values of TIL density, suggesting that larger tumors and, especially those with high ability to infiltrate adjacent normal structures, have often an immunologically cold microenvironment. The hypothesis that tumors reject their immune-cell component during growth is sound but impossible to confirm in human histopathological studies. Time-course biopsies are not feasible; patients receive therapy immediately after diagnosis. This inverse association of TIL density with T-stage has been confirmed in other studies in head neck cancer [27,28].

Using the median value, we grouped patients in two categories: low TIL density (cold tumors) vs. high TIL density (hot tumors). Analysis of TIL density and response to RT/chemo-RT showed no significant association, although there were higher numerical values in complete responders. In a study by Fiedler et al., high CD4+ TIL density was linked with higher complete response rates, while CD8+ TIL density was not [29]. This suggests that qualitative parameters of TILs may differentially affect response to RT. Nevertheless, locoregional post-RT progression-free survival was significantly worse in patients with low TIL density, a finding which has also been confirmed in a previous study by Balermpas et al. [30]. A study by Suzuki et al. also shows poor progression-free survival in patients with cold tumors, although this term has been used to describe overall disease failure (local and distant) [31]. Tumor regression following RT is mainly affected by tumor radiosensitivity, hypoxia conditions, and hypoxia-related intracellular molecular pathways. Despite the fact that the contribution of immune-mediated cell death during RT may be substantial, daily fractionated RT also kills the sensitive lymphocytes that are irradiated in tumors and lymph nodes. This could explain the lack of association between response and TILs. However, during the post-irradiation phase, residual tissue from originally hot tumors may become more intensively populated by lymphocytes, which empowers the post-irradiation immune-mediated tumor clearance. This explains the significant association between high TIL density and prolonged locoregional relapse-free intervals in univariate and multivariate analysis. The basis of this hypothesis is extensively discussed in one of our previous review articles [9]. High locoregional control, as expected, was followed by improved disease-specific overall survival, which is confirmed uniformly in published studies [27,28,29,30,31,32,33].

Another interesting finding was the significant association of the overexpression of three hypoxia and acidity-related markers, namely, HIF1α, HIF2α, and CA9, with an immunologically cold tumor microenvironment. This observation is explained by the well-known adverse effects of hypoxia and acidity on lymphocyte survival and proliferation [14,15]. Our group has confirmed a similar association in breast and lung cancer studies [22,34]. The pharmacological blockage of HIF or CA9 with specific inhibitors to transform the cold immune environment of such HIF-driven tumors could eventually have therapeutic implications in cancer immunotherapy. Experimental studies support this hypothesis. Luo et al. showed that treating mice bearing a syngeneic lung carcinoma with PX-478 (a selective HIF1α inhibitor) resulted in increased intratumoral accumulation of granzyme B secreting TILs, which prolonged the survival of mice treated with immunotherapy [35]. Jayaprakash et al. demonstrated that the hypoxia-activated prodrug TH-302 reduces hypoxia in spontaneous prostate cancer tumors and drives influx of T-cells, enhancing the efficacy of anti CTLA4/PD-L1 immunotherapy [36]. The treatment of mice with experimental tumors with SLC-0111, a small molecule inhibitor of CA9, reduced the glycolytic metabolism and extracellular acidification, decreased regulatory cell intratumoral accumulation, and increased the frequency of Th1 cells. Furthermore, SLC-0111, in combination with immune checkpoint inhibitors, significantly delayed tumor growth and the development of metastasis [37].

There are several limitations of the current study. The most obvious limitation is that the study included HNSCCs of different locations. This inhomogeneity may be a source of bias, provided that certain HNSCC locations are well known to link with a better prognosis. Chemotherapy has been administered to the vast majority of patients concurrently with irradiation, so the link between TIL density and the outcome of radiotherapy may have also been affected by the sensitivity to chemotherapy or even an eventual link between immunity and response to cetuximab. Another important issue that originates from the interesting herein reported link between poor TIL density and hypoxia is the strong contribution of the expression of hypoxia-related molecules to radio-resistance. Due to the low number of cases, such an analysis was not feasible in the current study, and larger samples are demanded to investigate the role of TIL density in hypoxic HNSCCs.

## 5. Conclusions

The findings of the current study strongly support the concept that tumor phenotypes allowing for the intratumoral accumulation of lymphocytes have a better outcome following radical RT/chemo-RT. Locoregional progression was significantly lower in originally pre-RT immunologically hot tumors. It is suggested that these tumors are susceptible to enhanced immunogenic post-irradiation tumor clearance due to their higher permissiveness to lymphocytic infiltration. Moreover, the provided evidence that the intratumorally activated HIF- and CA9-related pathways that characterize immunologically cold tumors highlight targets for therapeutic interventions. Such treatment policies could improve the efficacy of RT and immunotherapy, opening new windows for clinical trials in immuno-RT.

## Figures and Tables

**Table 1:** Patient and disease characteristics.

**No Patients**	**121**
**Age**	
Range	35–86
Median	66
**Performance Status**	
0	99 (81.8)
1	22 (18.2)
**Sex**	
Male	108 (89.2)
Female	13 (10.8)
**Primary Tumor Location**	
Larynx	57 (47.1)
Hypopharynx	9 (7.4)
Oropharynx	12 (9.9)
Oral cavity	15 (12.4)
Nasopharynx	13 (10.7)
Neck CUP *	9 (7.5)
Parotid	2 (1.7)
Lower Lip	4 (3.3)
**Differentiation**	
Grade 1	38 (31.5)
Grade 2	30 (24.9)
Grade 3	53 (43.6)

Number of patients followed by percentages in the brackets. * CUP = Cancer of Unknown Primary.

## Data Availability

All data are available in the files of the Department of Radiotherapy and Oncology and Department of Pathology, Democritus University of Thrace. The data presented in this study are available on request from the corresponding author. The data are not publicly available due to ethical reasons.

## Supplementary Materials

The following supporting information can be downloaded at: https://www.mdpi.com/article/10.3390/curroncol29060342/s1, Figure S1: Kaplan–Meier disease-specific overall survival of 48 patients stratified for: (a) TIL density and HIF1α expression, (b) TIL density and HIF2α expression, and (c) TIL density and CA9 expression. (TIL = tumor infiltrating lymphocytes).
