# Supplementary material for: Prognostic and Predictive Relevance of Tumor-Infiltrating Lymphocytes in Squamous Cell Head–Neck Cancer Patients Treated with Radical Radiotherapy/Chemo-Radiotherapy
Source: Curr Oncol. 2022 Jun 15;29(6):4274–84. doi: 10.3390/curroncol29060342 (PMC9222114; doi:10.3390/curroncol29060342)
Supplement: Supplementary file 1 [file curroncol-29-00342-s001.zip › curroncol-1754866-supplementary.pdf]

Current Oncology

MDPI

Article

Prognostic and Predictive Relevance of Tumor-Infiltrating Lymphocytes in Squamous Cell Head–Neck Cancer Patients Treated with Radical Radiotherapy/Chemo-Radiotherapy

Ioannis M. Koukourakis, Anastasia G. Gkegka, Erasmia Xanthopoulou, Christos Nanos, Alexandra Giatromanolaki and Michael I. Koukourakis

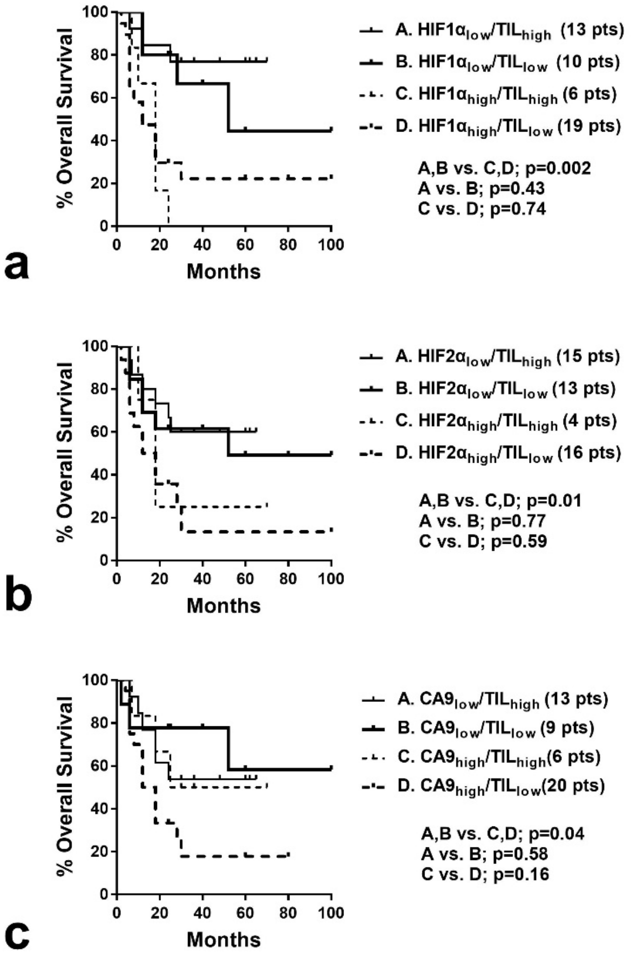

Figure S1. Kaplan–Meier disease-specific overall survival of 48 patients stratified for: (a) TIL density and HIF1α expression, (b) TIL density and HIF2α expression, and (c) TIL density and CA9 expression. (TIL = tumor infiltrating lymphocytes).
